# Discrepancy between PCR based SARS-CoV-2 tests suggests the need to re-evaluate diagnostic assays

**DOI:** 10.1186/s13104-021-05722-5

**Published:** 2021-08-17

**Authors:** Muhammad Zain Mushtaq, Sadia Shakoor, Akbar Kanji, Najma Shaheen, Asghar Nasir, Zeeshan Ansar, Imran Ahmed, Syed Faisal Mahmood, Rumina Hasan, Zahra Hasan

**Affiliations:** 1 Department of Medicine, Aga Khan University, Karachi, Pakistan; 2 Department of Pathology and Laboratory Medicine, Aga Khan University, Karachi, Pakistan; 3 Department of Pediatrics and Child Health, Division of Women and Child Health, Aga Khan University, Karachi, Pakistan

**Keywords:** SARS-CoV-2, PCR, COVID-19, Spike gene

## Abstract

**Objective:**

We investigated the discrepancy between clinical and PCR-based diagnosis of COVID-19. We compared results of ten patients with mild to severe COVID-19. Respiratory samples from all cases were tested on the Roche SARS-CoV-2 (Cobas) assay, Filmarray RP2.1 (bioMereiux) and TaqPath™ COVID19 (Thermofisher) PCR assays.

**Results:**

Laboratory records of ten patients with mild to severe COVID-19 were examined. Initially, respiratory samples from the patients were tested as negative on the SARS-CoV-2 Roche^**®**^ assay. Further investigation using the BIOFIRE^**®**^ Filmarray RP2.1 assay identified SARS-CoV-2 as the pathogen in all ten cases. To investigate possible discrepancies between PCR assays, additional testing was conducted using the TaqPath™ COVID19 PCR. Eight of ten samples were positive for SARS-CoV-2 on the TaqPath assay. Further, Spike gene target failures (SGTF) were identified in three of these eight cases. Discrepancy between the three PCR assays could be due to variation in PCR efficiencies of the amplification reactions or, variation at primer binding sites. Strains with SGTF indicate the presence of new SARS-CoV-2 variant strains. Regular modification of gene targets in diagnostic assays may be necessary to maintain robustness and accuracy of SARS-CoV-2 diagnostic assays to avoid reduced case detection, under-surveillance, and missed opportunities for control.

**Supplementary Information:**

The online version contains supplementary material available at 10.1186/s13104-021-05722-5.

## Introduction

Robust diagnostic testing for SARS-CoV-2 is integral to disease surveillance and control of COVID-19. Reverse-transcription polymerase chain reaction (RT-PCR) based assays are the most widely used assay to detect RNA viruses. Selection of target genes for diagnostic assays is critical and most diagnostic assays in use include two or more gene targets to maximize diagnostic accuracy (sensitivity and specificity) [1].

SARS-CoV-2 was identified in December 2019 as a beta-coronavirus of the *Sarbecovirus* family, with a positive-sense RNA genome of 29.9 kb in size, fourteen open reading frames (*orf*) encoding accessory nonstructural viral proteins, the nucleocapsid (N), membrane (M), spike (S), and envelope (E) structural proteins [2]. In order for SARS-CoV-2 testing to be reliable, it is necessary that assays that identify viral RNA should be consistent and comparable. When recommendations for SARS-CoV-2 diagnostics were first made, the E gene and RdRp were amongst recommended potential target geness [3]. The E gene is known to have higher sensitivity while the inclusion of the N, S, and RNA dependent RNA polymerase (*RdRp*) genes are recommended for higher specificity [4].

No test is 100% accurate and false-negative results with commercially available diagnostic assays have been documented since the early days of the pandemic [5]. False-negative results in the context of symptomatic COVID-19 illness may have several determinants, such as the clinical specimen type (sputum and bronchoalveolar lavage have higher detection rates than nasal and naso- or oropharyngeal specimens) [2], temporal variation in viral shedding [6], as well as diagnostic primer/probe mismatches with infecting SARS-CoV2 virus sequence [7]. However, in the context of increasing reports of SARS-CoV-2 variants seeing false negative results in diagnostic tests are of particular concern, as there is a wider implication on misidentification of asymptomatic cases as well, particularly [8, 9].

## Main text

### Methods

Patient specimens were collected from cases admitted into the COVID-19 unit at The Aga Khan University Hospital, Karachi, Pakistan. Inclusion criteria cases were those who had a high clinical suspicion for COVID-19 but had a negative SARS-CoV-2 PCR test conducted at the time of their hospital admission using the SARS-CoV-2 Cobas^®^ 6800 assay (Roche diagnostics Rotkreuz, Switzerland). The following were considered as signs of COVID-19: classical presentation in terms of signs and symptoms, need for supplemental oxygen support, deranged inflammatory parameters and absence of alternative diagnosis. Exclusion criteria were cases with the abovementioned clinical signs who had an alternative cause for their respiratory illness and those who had a positive SARS-CoV-2 PCR results.

Patients were diagnosed with COVID-19 based on clinical, laboratory and radiological parameters which were used to assess the severity of disease. Clinical parameters included physical signs like tachypnea, tachycardia, hypoxia (SpO_2_ < 94% at room air), while laboratory investigations included hypoxemia (PaO_2_ < 80 mm Hg), hyperferritinemia, raised LDH and CRP. Radiologically, patients were assessed on the basis of Computerised tomography (CT) scan or X-ray chest.

COVID-19 severity was assessed on the basis of need for supplemental oxygen support, raised levels of inflammatory markers and more than 50% involvement of lungs on a CT scan or X- ray chest. Severity was ranked as per the WHO ordinal scale [10]. In each case, nasopharyngeal swab specimens were first tested for SARS-CoV-2 RNA using the SARS-CoV-2 Cobas^®^ (Roche diagnostics Rotkreuz, Switzerland) targeting *orf1-ab* and E genes. In the case that the SARS-CoV-2 Roche RT-PCR was found to be negative, a second PCR was conducted using the BIOFIRE^**®**^ Filmarray RP2.1 test (bioMereiux, Marcy-l’Étoile, France) which includes four bacterial and 18 viral pathogen targets i.e. Adenovirus, influenza A viruses H1, 2009H1, H3 (FluA-H1, FluA-2009H1, FluA-H3), influenza B virus (FluB), parainfluenza virus types 1–4 (Para 1–4), coronaviruses 229E, HKU1, OC43, and NL63 (CoV-HKU1, NL63, 229E, OC43), MERS-CoV (MERS coronavirus), human metapneumovirus (hMPV), Respiratory Syncytial Virus (RSV), human rhinovirus/enterovirus (Rhino/Entero), *Chlamydia pneumoniae*, *Mycoplasma pneumoniae*, *Bordetella pertussis*, and *Bordetella parapertussis* in addition to SARS-CoV2 (S and M gene gene targets). All the RT-PCR assays were conducted at the Aga Khan University, Karachi. SARS-CoV-2 Cobas^®^ and BIOFIRE^**®**^ Filmarray RP2.1 tests were conducted at the AKUH Clinical Laboratories which are accredited by the College of American Pathologists. For further investigation of discrepancies, a third PCR was conducted using the TaqPath™ COVID19 (Thermo, Applied Biosystems, USA) assay (S, N and *orf1ab* gene targets) at the AKU Research Laboratory. Assay details are provided in Additional file 1.

### Results

We report on ten patients with COVID-19 from Karachi, Pakistan from 18th January to 18th February 2021, presenting to acute care at a tertiary hospital. The patients were investigated for COVID-19 based on their clinical presentation. They were mostly aged 65–74 years (60%), followed by two cases aged > 75 years and two below 54 years of age, Table 1. Patients had between five and forty days of illness with a median period of 8 days. Laboratory parameters found to be raised in all cases were: hyperferritinemia, high biomarker levels (C-reactive protein, D-dimer), details not shown. Chest imaging showed ground-glass opacities or bilateral parenchymal infiltrates, and high population prevalence in all cases. Respiratory samples tested by PCR for SARS-CoV-2 using the SARS-CoV-2 Cobas Roche assay were negative in all cases. Due to a high clinical suspicion for COVID-19, samples were further set for testing on the Filmarray RP2.1 platform. In all ten cases, Filmarray results indicated SARS-CoV-2 RNA to be present in the respiratory samples.

**Table 1:** Description of COVID-19 patients and their differential diagnostic PCR results

No	Age range^a^ (years)	Duration of illness at presentation (Days)	Severity	Chest imaging (radiography/ CT scan)	Results—cobas 6800 SARS-Co-V-2 assay (***orf1ab*** and **E** gene targets)	Results for the BIOFIRE Filmarray RP2.1 assay (**S** and **M** gene targets)^d^	Results for TaqPath 2019-nCoV assay ***orf1ab***, **S** and **N** gene targets with (CT values)	Explanation for discrepancy with cobas 6800 Roche or BIOFIRE Filmarray assays
1	> 75	7	Non-severe	CT: bilateral ground glass opacities	NEG^b^	POS	*orf1ab* (25.86), S (18.49), and N (27.95) detected	Possible *orf1ab* and E gene polymorphism
2	65–74	5	Severe	CT: bilateral patchy consolidations	NEG^b^	POS^c^	*orf1ab* (30.11) and N (30) detected (SGTF)	Possible *orf1ab,* E and S gene polymorphism
3	65–74	8	Severe	CT: bilateral ground glass opacities	NEG^b^	POS	No targets detected, SGTF	Possible N, E, and *orf1ab* gene and S gene polymorphism
4	45–54	5	Severe	CT: bilateral ground glass opacities	NEG^b^	POS^c^	S (30.63) and N (31.14) detected *(orf1ab* not detected)	Possible E and *orf1ab* polymorphism
5	65–74	8	Critical	Radiograph: bilateral infiltrates	NEG^b^	POS	*orf1ab* (11.85), S (9.91), and N (19.02) detected	Possible *orf1ab* and E gene polymorphism
6	65–74	10	Critical	Radiograph: predominant right infiltrates	NEG^b^	POS	*orf1ab* (18.16), S (15.53), and N (18.45) detected	Possible *orf1ab* and E gene polymorphism
7	65–74	40	Long COVID/Post COVID sequelae	CT: bilateral ground glass opacities	NEG^b^	POS	*orf1ab* (21.15), S (23.85), and N (31.71) detected	Possible *orf1ab* and E gene polymorphism
8	> 75	8	Critical	Radiograph: bilateral infiltrates	NEG^b^	POS	*orf1ab* (31.2), S (31.41), and N (31.22) detected	Possible *orf1ab* gene polymorphism
9	65–74	7	Critical	CT: bilateral ground glass opacities	NEG^b^	POS	No targets detected, SGTF	Possible N, E, *orf1ab and S gene* polymorphism
10	35–44	10	Non-severe	CT: bilateral ground glass opacities	NEG^b^	POS^c^	*orf1ab* (18.92), S (24.97), and N (20.68) detected	Possible *orf1ab* and E gene polymorphism

*NP* not performed, *D* Detected, *NEG* Negative, *POS* Positive, *CT* Cycle threshold, *SGTF* S gene target failure

^a^Patient ages are given as age ranges to preserve confidentiality and anonymity

^b^Results rechecked on a new sample collected within 48 h

^c^Results rechecked on same sample

^d^Results negative for all other pathogens detected by Filmarray RP2.1 i.e., Adenovirus, influenza A viruses H1, 2009H1, H3 (FluA-H1, FluA-2009H1, FluA-H3), influenza B virus (FluB), parainfluenza virus types 1 to 4 (Para 1–4), coronaviruses 229E, HKU1, OC43, and NL63 (CoV-HKU1, NL63, 229E, OC43), MERS-CoV (MERS coronavirus), human metapneumovirus (hMPV), Respiratory Syncytial Virus (RSV), human rhinovirus/enterovirus (Rhino/Entero), *Chlamydia pneumoniae*, *Mycoplasma pneumoniae*, *Bordetella pertussis*, and *Bordetella parapertussis*

To further investigate this, the same ten respiratory specimens tested by Filmarray test were subsequent tested for the presence of SARS-CoV-2 RNA on the TaqPath™ COVID19 assay. Here we found, eight respiratory specimens to be positive and two were negative (Table 1). All three gene targets in the TaqPath assay (Orf1ab, N and S) were detected in six samples. In one sample N and S were detected but Orf1ab target amplification was absent. S gene target failure (SGTF) was identified in three specimens. The CT values of gene targets amplified in the TaqPath assay were CT 31 and below indicating, high to medium viral loads of SARS-CoV-2 [11].

### Discussion

Our data suggest show variations between diagnostic platforms for SARS-CoV-2. A summary of differential commercial RT-PCR test results and possible explanations are given in Table 1. Single nucleotide mismatch in primer/ probe, especially in the 3′ binding region, may result in failure of target binding and false-negative results [6]. The *﻿orf﻿1ab*, N, and S regions are shown to be the most mutable in SARS-CoV-2, whilst E gene and M gene have been reported to be relatively less error prone [7]. Genomic sequencing would be necessary to further delineate the assumed polymorphisms in SARS-CoV-2 *orf1ab,* N, E, and S genes that could result in the discrepancies identified. However, as sequences of target regions in commercial assays are proprietary, a direct comparison between the binding regions of *orf1ab* in the Roche and TaqPath assays; and of the S region in the Filmarray and TaqPath assays cannot be made.

The TaqPath COVID19 assay has been used to screen for UK Variant of Concern (B.1.1.7) with SGTF used as a surrogate marker for H69-V70 variant detection [8]. We have limited data on genomic surveillance of SARS-CoV-2 in Pakistan however, we have recently identified the introduction of B.1.1.7 lineage strains (unpublished data, sequence submitted). The variability observed in the three cases with S gene drop-out suggests the presence of new variants. Importantly, given the CT values of the gene targets detected in each case by the TaqPath assay, the samples had a medium to high viral load. Therefore, it is unlikely that discrepancy between SARS-CoV-2 diagnosis between assays was due to variation between assay sensitivity, further supporting our hypothesis.

These cases we present illustrate the importance of taking into account both the quality of the diagnostic assay and its appropriate design strategy to best capture SARS-CoV-2 strains moving forward. This report is a problem statement and has not made any comparisons or tested any hypotheses. We suggest that the issue of diagnostic test discrepancy be further studied through systematic research. While keeping primer and probe information proprietary is the norm, the medical and research community will benefit from requests from scientists to reveal further information, which is often not made available even upon request [12]. We propose frequent evaluation of national databases of viral genome sequences to inform standards on diagnostic assays. Such an initiative can advise on targets with low mutation frequency, such that > 99% of circulating variants are detectable using selected primers and probes. Further, in keeping with the rapidly evolving nature of the SARS-CoV-2 genome it is of particular consequence that there be a regular review of the target primers being used and an improvement of diagnostic assays to keep up the expected sensitivity and specificity required of diagnostic assays for COVID-19.

## Limitations

Our results are subject to inherent limitation due to small sample size of the study. Further, as we do not have genome sequences available of the SARS-CoV-2 strains described in the study, we cannot ascertain the polymorphisms present in the genomes. Further, due to the proprietary nature of the commercial assays used it would not be possible to associate sequence variations with differential amplification frequencies of the PCR tests.

## Supplementary Information

**Additional file 1.** Description of PCR tests.

## Data Availability

The data is available in the table here and raw data is available upon request.

## Abbreviations

COVID-1: disease caused by novel coronavirus 2019
CRP: C-reactive protein
CT: Cycle Threshold
CT scan: Computerized tomography scan
D: Detected
E: Envelope
FluB: Influenza B virus
FluA: Influenza A virus
LDH: Lactate dehydrogenase
MERS–CoV: MERS coronavirus
hMPV: Human metapneumovirus
NP: Not Performed
NEG: Negative
N: Nucleocapsid
M: Membrane
OrF: open reading frame
Para (1-4): Influenza virus types para (1-4)
POS: Positive
PCR: Polymerase Chain Reaction
RSV: Respiratory Synctial virus
Rhino/Entero: Human rhinovirus/enterovirus
RdRp: RNA dependent RNA polymerase
RT-PCR: Reverse transcriptase polymerase chain reaction
SARS-CoV-2: Severe acute respiratory syndrome coronavirus 2
S: Spike
SGTF: S gene target failure
WHO: World Health Organization
