# Supplementary material for: Discrepancy between PCR based SARS-CoV-2 tests suggests the need to re-evaluate diagnostic assays
Source: BMC Res Notes. 2021 Aug 17;14:316. doi: 10.1186/s13104-021-05722-5 (PMC8369441; doi:10.1186/s13104-021-05722-5)
Supplement: Supplementary file 1 — Additional file 1. Description of PCR tests. [file 13104_2021_5722_MOESM1_ESM.docx]

**Additional file 1. Description of PCR tests.**

SARS-CoV-2 RT-PCR methods employed:

Diagnostic testing of nasopharyngeal swab specimens for SARS-CoV-2 RNA was conducted by reverse transcription (RT) polymerase chain reaction (PCR) at the Clinical Laboratories, Aga Khan University Hospital (AKUH), Karachi, Pakistan.

Testing for SARS-CoV-2 using the SARS-CoV-2 Cobas® 6800 assay (Roche diagnostics Rotkreuz, Switzerland) is a onestep method. In this 700 ul of respiratory swab sample is directly added to a secondary tube that is loaded into the Cobas® system, Roche Diagnostics. RNA extraction is directly followed by targeted RT-PCR for *orf1ab* and E gene targets. Amplification data and CT values of *orf1ab* and E gene targets were available.

# Testing for SARS-CoV-2 using the BIOFIRE^®^Respiratory 2.1 Filmarray RP2.1 assay (bioMereiux, Marcy-l'Étoile, France) is a one-step method. In this 1000 ul of respiratory swab sample is directly added to the testing cartridge which is loaded into the BIOFIRE^®^Filmarray system. RNA extraction is directly followed by targeted RT-PCR for 4 bacterial and 18 viral pathogen targets i.e. Adenovirus, influenza A viruses H1, 2009H1, H3 (FluA-H1, FluA-2009H1, FluA-H3), influenza B virus (FluB), parainfluenza virus types 1 to 4 (Para 1–4), coronaviruses 229E, HKU1, OC43, and NL63 (CoV-HKU1, NL63, 229E, OC43), MERS-CoV (MERS coronavirus), human metapneumovirus (hMPV), Respiratory Syncytial Virus (RSV), human rhinovirus/enterovirus (Rhino/Entero), *Chlamydia pneumoniae*, *Mycoplasma pneumoniae*, *Bordetella pertussis*, and *Bordetella parapertussis* in addition to SARS-CoV2 (S and M gene gene targets). Results of the assay are directly transmitted to the laboratory management system. *(Note: CT values are not available in this system)*

Testing for SARS-CoV-2 using the TaqPath ^TM^ COVID-19 (Thermo, Applied Biosystems, USA) assay is a two-step method. First, one hundred and forty microliters of respiratory swab specimen were used for RNA extraction using the QIAAmp RNA minikit (Qiagen, USA) using the manufacturer’s recommendation. RT-PCR was set up using 10 ul of RNA using the TaqPath ^TM^ COVID19 PCR assay as per the manufacturer’s recommendations using the Applied Biosystems QuantStudio 5 Real-Time PCR System. Amplification data and Ct values of *orf1ab,* N and S gene targets were available.
